# Circadian Syndrome Mediates the Association Between Body Roundness Index and Incident Stroke Risk in Middle‐Aged and Older Chinese Adults: A Nationwide Prospective Cohort Study

**DOI:** 10.1155/bn/1282354

**Published:** 2026-03-27

**Authors:** Gaiying Ma, Hongwei Liu, Haiyan Wu, Minheng Zhang, Yonglai Zhang, Haixia Fan, Ke Shi

**Affiliations:** ^1^ Department of Neurology, Peking University First Hospital Taiyuan Branch (Taiyuan Central Hospital of Shanxi Medical University), Taiyuan, Shanxi Province, China; ^2^ Department of Neurology, Xuanwu Hospital, Capital Medical University, Beijing, China, ccmu.edu.cn; ^3^ Department of Sleep Center, First Hospital of Shanxi Medical University, Taiyuan, Shanxi Province, China, sxmu.edu.cn; ^4^ Department of Gerontology, The First People′s Hospital of Jinzhong, Jinzhong, Shanxi Province, China; ^5^ School of Software, North University of China, Taiyuan, Shanxi Province, China, nuc.edu.cn; ^6^ Department of Neurology, Xiangshui County People′s Hospital, Yancheng, Jiangsu Province, China

**Keywords:** anthropometric index, body roundness index, CHARLS, circadian syndrome, stroke

## Abstract

**Background:**

Despite BRI being a novel measure for chronic disease risk, the relationship between BRI and stroke risk under varying circadian syndrome (CircS) conditions is still unclear.

**Methods:**

The analysis utilized data from 7535 participants involved in the China Health and Retirement Longitudinal Study (CHARLS). CircS was defined by at least four concurrent metabolic disturbances due to circadian rhythm disruption. To ensure robustness, the BRI‐stroke association was assessed using Cox models, restricted cubic splines, Kaplan–Meier curves, and mediation analyses, along with subgroup and sensitivity analyses. The receiver operating characteristic (ROC) analysis was used to compare the stroke risk prediction performance of BRI with conventional metabolic indices.

**Results:**

Following full adjustment, higher log (BRI) values were significantly associated with an increased risk of stroke (HR = 2.50, 95% CI: 1.81–3.44, *p* < 0.001), with Q4 showing a greater risk than Q1 (HR = 2.23, 95% CI: 1.63–3.04, *p* < 0.001). In CircS patients, no significant association was observed (log (BRI) HR = 1.23, 95% CI: 0.55–2.76; Q4 vs. Q1 HR = 0.61, 95% CI: 0.21–1.77; all *p* > 0.05). However, the association remained significant in non‐CircS individuals (log (BRI) HR = 2.36, 95% CI: 1.61–3.47; Q4 vs. Q1 HR = 2.08, 95% CI: 1.46–2.98; all *p* < 0.001). Subgroup and sensitivity analyses bolstered the robustness of our findings. CircS mediated 21.0% of the association between BRI and stroke risk (95% CI: 18%–91%, *p* < 0.001). With an area under the curve (AUC) of 0.656, BRI demonstrated moderate predictive capability for stroke, outperforming other anthropometric indices.

**Conclusions:**

An increased BRI is associated with a higher stroke risk, partially mediated by CircS, highlighting the potential role of circadian health in stroke prevention.

## 1. Introduction

With the aging population and the growing prevalence of cardiometabolic disorders, the global impact of stroke, a leading cause of mortality and disability‐adjusted life years (DALYs), is expected to escalate [1]. Even though hypertension, diabetes, and dyslipidemia are acknowledged as traditional risk factors, there is an increasing interest in innovative and modifiable predictors that may enhance early stroke detection and prevention [2, 3].

Central obesity has been closely associated with an increased risk of stroke [4]. Conventional anthropometric measures, such as body mass index (BMI) and waist circumference (WC), have inherent limitations in accurately capturing visceral adiposity and body fat distribution [5]. To improve upon these limitations, the body roundness index (BRI) was introduced as a geometric measure that combines WC and height to provide a better approximation of body shape and visceral fat accumulation [6]. In the adult population of China, BRI has been shown to be a reliable predictor of chronic diseases and is useful for evaluating long‐term health risks and outcomes [7, 8]. Moreover, accumulating evidence suggests that BRI may outperform traditional adiposity indices in predicting cardiovascular and metabolic diseases [7, 9, 10]. Nonetheless, the link between BRI and the occurrence of stroke is not well defined, especially in large‐scale prospective research.

Recent research has brought attention to the critical role of circadian syndrome (CircS) in metabolic health [11]. The circadian system, made up of the central clock in the suprachiasmatic nucleus and peripheral clocks in multiple tissues, plays a key role in the regulation of metabolic processes. Disturbances in this system can lead to impaired glucose metabolism, which in turn raises the risk of Type 2 diabetes and other metabolic disorders [12, 13]. This interaction is bidirectional: circadian misalignment can negatively affect metabolic functions, and conditions such as obesity and metabolic syndrome can disturb circadian rhythms, perpetuating a cycle of dysfunction [14, 15]. Furthermore, CircS has been associated with an increased risk of cardiovascular events, including stroke [16, 17], and exerts a multifaceted influence on cardiovascular disease risk [18], playing a critical role in its development and progression. However, the interaction between CircS and newer adiposity indices, such as the BRI, and how this relationship influences stroke risk remains poorly understood.

Previous studies have primarily focused on the individual effects of risk factors such as hypertension, hyperglycemia, and dyslipidemia [18–22]. However, the potential overlap and interactions between these factors have not been fully explored. Moreover, the presence of multiple components of CircS, rather than just one, adds complexity to stroke risk assessment. The combined effects of metabolic abnormalities, including hypertension, hyperglycemia, dyslipidemia, sleep disturbances, and depressive symptoms, may either counterbalance or amplify each other. This underscores the need for a more comprehensive evaluation of the cumulative impact of CircS on stroke risk. In spite of these insights, the involvement of CircS in the relationship between BRI and the risk of stroke remains insufficiently examined. To address these gaps, we conducted a prospective cohort study using data from the China Health and Retirement Longitudinal Study (CHARLS) [23]. The primary objectives of this study were as follows: (1) examine the association between baseline BRI and new‐onset stroke; (2) assess the potential modifying effect of CircS; (3) explore whether CircS explains the relationship between high BRI and subsequent stroke risk; (4) evaluate the contributions of CircS to the indirect pathway and determine whether its simultaneous occurrence amplifies risk through compounded disruptions in circadian regulation; and (5) assess the predictive performance of BRI relative to traditional anthropometric measures. Our findings are aimed at informing stroke risk assessment strategies based on body shape and enhancing the early identification of individuals at elevated risk.

## 2. Method

### 2.1. Study Design and Population

**Figure 1:**
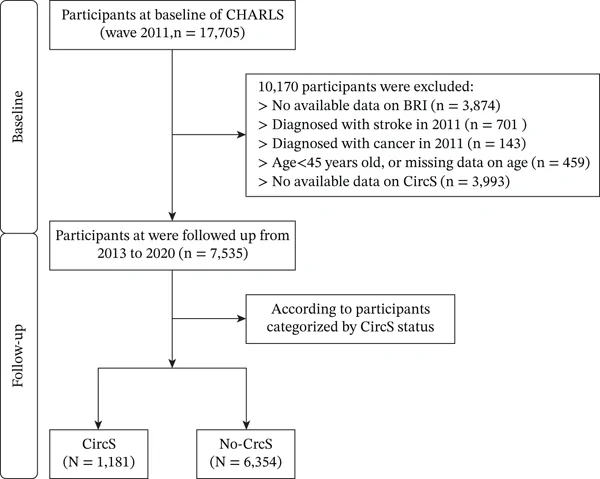
Flow chart of participants selection. Abbreviations: CHARLS, China Health and Retirement Longitudinal Study; BRI, body roundness index; CircS, circadian syndrome.

Data used in this research were drawn from the 2011 baseline survey of the CHARLS, which included 17,705 participants. A total of 10,170 participants were excluded for the following reasons: 3874 lacked BRI data, 701 had a self‐reported stroke diagnosis in 2011, 143 had cancer diagnosed in 2011, 459 were younger than 45 years or had missing age data, and 3993 had no available data on CircS. The 7535 remaining participants were followed over the period from 2013 to 2020. Based on CircS status, participants were divided into two groups: 1181 individuals in the CircS group and 6354 individuals in the no‐CircS group (Figure 1).

### 2.2. Measurements of BRI

BRI, calculated from WC and height, is a geometric index created to evaluate total body fat and visceral adiposity, adjusting for height without relying on body weight or hip circumference [6, 24]. Using standardized protocols, height and WC were measured in meters at the mobile examination center to ensure data consistency and accuracy. These measurements were then utilized to determine the BRI for each participant. The BRI is calculated through the following formula: BRI = 364.2–365.5 × √[(WC/(2*π* × 0.5 × height)^2^].

### 2.3. Measurements of CircS

CircS refers to a cluster of co‐occurring metabolic abnormalities and disruptions in circadian regulation. It is clinically defined by the presence of at least four of the following seven components: central obesity (WC ≥ 80 cm for women or ≥ 85 cm for men); elevated triglycerides (TGs) (≥ 150 mg/dL or current use of lipid‐lowering medications); low–high‐density lipoprotein cholesterol (HDL‐C < 50 mg/dL for women or < 40 mg/dL for men, or pharmacological treatment thereof); raised blood pressure (systolic ≥ 130 mmHg, diastolic ≥ 85 mmHg, or antihypertensive therapy); elevated fasting glucose (≥100 mg/dL or use of glucose‐lowering agents); insufficient sleep duration (fewer than 6 h per day); and the presence of depressive symptoms, typically evaluated using validated instruments such as the Patient Health Questionnaire‐9 (PHQ‐9 ≥ 10) [16, 25–27].

### 2.4. Covariates

Demographic variables included age, sex (male or female), marital status (categorized as married/cohabiting with a spouse versus other), and educational attainment (classified as junior high school or below, senior high school, and college or above). Behavior‐related factors included whether someone smoked (never, formerly, or currently) and their drinking status (never, formerly, or currently). The patient′s records detailed whether they had heart disease, hypertension, lipid imbalances and their weight classification (normal, overweight, or obese). Finally, the lab evaluations included markers for kidney function, which were serum creatinine and blood urea nitrogen.

### 2.5. Outcome Identification

The outcome of this study was new‐onset stroke, identified based on self‐reported doctor diagnoses during the follow‐up surveys, which began in 2013 and continued through 2020. Participants initially without a stroke history who reported a stroke during the follow‐up were categorized as having experienced a new stroke event. Information about strokes was collected by using the following standardized questions [28, 29]: (1) Has a doctor advised you that you experienced a stroke? (2) When did you first learn about or get a diagnosis for the stroke? and (3) Are you receiving any follow‐up treatment? The participant′s self‐reported positive responses pointed to a first stroke diagnosis, and the onset date was documented. To calculate the time of onset, the difference between the stroke onset date and the baseline survey date was used.

### 2.6. Statistical Analysis

Using R Version 4.4.1, statistical analyses were carried out, with a two‐sided *p* value of < 0.05 regarded as statistically significant. Continuous variables with distributions that were not normal were summarized using medians and interquartile ranges (Q1 and Q3), whereas frequencies and proportions were used for categorical variables. The Kruskal–Wallis rank‐sum test was employed for continuous variables that did not follow a normal distribution, and the chi‐square test was used for categorical variables in the group analysis. Multiple imputation via the mice package was employed to handle missing data, with their distribution outlined in Table S1.

Cox proportional hazards regression was performed to evaluate the association between BRI and stroke incidence, with hazard ratios (HRs) and 95% confidence intervals (CIs) reported. Covariates were chosen in advance, guided by biological plausibility and previous research. Scaled Schoenfeld residuals were used to test the proportional hazards assumption globally and for each covariate, revealing no significant violations (*p* > 0.05 for all covariates). We performed a variance inflation factor (VIF) analysis on all covariates in the models to evaluate multicollinearity. Table S2 reveals that all VIF values were under the threshold of three, suggesting that multicollinearity was not a concern and the estimates remained stable across the models. By applying a logarithmic transformation, the significant skewness in the BRI distribution was addressed, leading to better model convergence and fit. Using Cox proportional hazards regression, the connection between BRI and the risk of a new stroke was examined across three models with successive adjustments. Model 1 included no covariate adjustments, Model 2 accounted for demographic and behavioral factors (age, sex, marital status, smoking status, educational level, and drinking status), and Model 3 further adjusted for clinical and biochemical covariates, such as history of heart disease, hypertension, dyslipidemia, obesity status, serum creatinine, and blood urea nitrogen. The investigation applied restricted cubic spline (RCS) functions with three knots, selected using the Akaike information criterion (AIC), to analyze potential nonlinear associations between BRI and stroke risk, with adjustments for all covariates. The Kaplan–Meier estimator determined cumulative stroke incidence, stratified by CircS status, with group differences assessed via the log‐rank test. Subgroup analyses were carried out based on gender, age classification (< 60 vs. ≥ 60 years), presence of heart disease, smoking status, and alcohol consumption. Potential interaction effects were assessed on a multiplicative scale. The robustness of the main conclusions was assessed through two sensitivity analyses. First, the association between BRI and stroke risk was re‐evaluated using datasets that included participants with missing data. Second, to mitigate potential reverse causality, individuals who had a stroke within 2 years of baseline were excluded. The mma package in R was used to perform multiple mediation analysis to determine how CircS mediates the influence of BRI on the risk of stroke. This method allows for the simultaneous evaluation of multiple mediators. Two algorithms were employed to calculate the path coefficients, which included the direct effect (DE) of BRI on stroke, the indirect effect (IE) of BRI via CircS, and the total effect (TE) of BRI on the occurrence of stroke. The first algorithm involved calculating the TE of BRI on stroke, illustrating the complete impact of BRI on stroke risk. The IE shows the part of BRI′s effect on stroke that is mediated by CircS. In the second algorithm, the DE of BRI on stroke was estimated without CircS mediation, using samples from the first algorithm and random permutation methods. The proportion of the indirect effect in the total effect (IE/TE) indicates the degree to which CircS mediates the association between BRI and stroke risk. To confirm the mediation effects, TE must be significant; if TE is close to zero (*p* > 0.05), relative effects (REs) are not reported. Based on earlier research and theoretical importance, we controlled for potential confounders in the BRI‐CircS, CircS‐stroke, and BRI‐stroke associations. Moreover, we ensured that the mediation pathways remained unaffected by unmeasured confounders by using a covariate matrix to control for potential confounding influences, incorporating variables based on prior research and theoretical relevance. To strengthen the robustness of the mediation model, we checked for any interaction between the exposure (BRI) and the mediator (CircS) to ensure it did not alter the mediation effect. Monte Carlo resampling with *n* = 50 and *n*_2_ = 100 iterations was used to improve the stability of the estimates. By excluding the WC component, sensitivity analyses were carried out to assess the potential impact of structural overlap between BRI and CircS. Mediation models were re‐estimated under this alternative specification to evaluate the robustness of the IE. Using receiver operating characteristic (ROC) curve analysis, model discrimination was assessed, with area under the curve (AUC) acting as the summary indicator of predictive accuracy. An AUC > 0.6 was considered indicative of adequate discriminatory ability. The reports also included sensitivity, specificity, positive predictive value (PPV), and negative predictive value (NPV) for various BRI cutoff points.

## 3. Results

### 3.1. Baseline Characteristics of Participants

The participant pool consisted of 7535 people, divided into 1181 in the CircS group and 6354 in the no‐CircS group. Table 1 displays an overview of the baseline demographic and clinical features.

**Table 1:** Baseline characteristics of participants categorized by CircS status.

Characteristic	Overall (*N* = 7535)	CircS status		*p* ^a^
		CircS (*N* = 1181)	No‐CircS (*N* = 6354)	
Age, median (Q1, Q3)	59.00 (52.00, 65.00)	60.00 (54.00, 66.00)	58.00 (52.00, 65.00)	< 0.001
BRI, median (Q1, Q3)	4.04 (3.23, 5.09)	5.13 (4.40, 6.02)	3.83 (3.10, 4.85)	< 0.001
Gender, *n* (%)				< 0.001
Male	3534.00 (46.90%)	323.00 (27.35%)	3211.00 (50.54%)	
Female	4001.00 (53.10%)	858.00 (72.65%)	3143.00 (49.46%)	
Marital status, *n* (%)				< 0.001
Married and living with spouse	6625.00 (87.92%)	991.00 (83.91%)	5634.00 (88.67%)	
Other	910.00 (12.08%)	190.00 (16.09%)	720.00 (11.33%)	
Education level, *n* (%)				< 0.001
Junior high school and below	5242.00 (69.57%)	911.00 (77.14%)	4331.00 (68.16%)	
Senior high school	2182.00 (28.96%)	257.00 (21.76%)	1925.00 (30.30%)	
College and above	111.00 (1.47%)	13.00 (1.10%)	98.00 (1.54%)	
Smoking status, *n* (%)				< 0.001
Never smoked	4557.00 (60.48%)	875.00 (74.09%)	3682.00 (57.95%)	
Currently smoking	2321.00 (30.80%)	209.00 (17.70%)	2112.00 (33.24%)	
Ever smoked	657.00 (8.72%)	97.00 (8.21%)	560.00 (8.81%)	
Drinking status, *n* (%)				< 0.001
Never drink	5248.00 (69.65%)	955.00 (80.86%)	4293.00 (67.56%)	
Currently drinking	1900.00 (25.22%)	163.00 (13.80%)	1737.00 (27.34%)	
Ever drunk	387.00 (5.14%)	63.00 (5.33%)	324.00 (5.10%)	
SBP, mmHg, median (Q1, Q3)	127.33 (114.67, 142.00)	138.33 (124.00, 153.33)	125.33 (113.67, 139.33)	< 0.001
DBP, mmHg, median (Q1, Q3)	75.00 (67.33, 83.33)	79.67 (72.00, 88.00)	74.00 (66.67, 82.00)	< 0.001
Heart rate, bpm, median (Q1, Q3)	72.00 (65.00, 79.00)	74.00 (67.00, 80.00)	72.00 (65.00, 79.00)	< 0.001
Waist circumference, cm, median (Q1, Q3)	84.90 (78.00, 92.00)	92.00 (86.80, 98.00)	83.00 (77.00, 90.20)	< 0.001
WHtR, median (Q1, Q3)	0.53 (0.49, 0.58)	0.59 (0.55, 0.63)	0.52 (0.49, 0.57)	< 0.001
ABSI, median (Q1, Q3)	0.83 (0.80, 0.86)	0.85 (0.81, 0.88)	0.82 (0.79, 0.86)	< 0.001
VAI, median (Q1, Q3)	7.07 (4.27, 12.33)	17.16 (11.38, 25.64)	6.18 (3.93, 10.00)	< 0.001
TC,, mg/dL, median (Q1, Q3)	191.37 (168.17, 216.50)	200.26 (175.13, 226.16)	190.21 (167.01, 214.56)	< 0.001
TG,, mg/dL, median (Q1, Q3)	103.54 (74.34, 150.45)	175.23 (128.32, 242.49)	95.58 (70.80, 133.63)	< 0.001
HDL‐C, mg/dL, median (Q1, Q3)	49.48 (40.59, 60.31)	39.05 (33.63, 45.23)	52.19 (42.91, 62.24)	< 0.001
LDL‐C, mg/dL, median (Q1, Q3)	115.21 (94.33, 138.02)	117.14 (93.17, 141.11)	114.82 (94.72, 137.63)	0.559
FBG, mg/dL, median (Q1, Q3)	102.24 (94.50, 112.32)	109.62 (99.72, 127.44)	101.34 (93.96, 110.34)	< 0.001
HbA1c, median (Q1, Q3)	5.20 (4.90, 5.40)	5.30 (5.00, 5.80)	5.10 (4.90, 5.40)	< 0.001
Serum creatinine, mg/dL, median (Q1, Q3)	0.76 (0.66, 0.88)	0.75 (0.64, 0.87)	0.76 (0.66, 0.88)	0.002
Blood urea nitrogen, mg/dL, median (Q1, Q3)	15.15 (12.58, 18.21)	14.62 (12.38, 17.31)	15.27 (12.60, 18.32)	< 0.001
Obesity status, *n* (%)				< 0.001
Normal weight	4452.00 (59.08%)	372.00 (31.50%)	4080.00 (64.21%)	
Overweight	2217.00 (29.42%)	513.00 (43.44%)	1704.00 (26.82%)	
Obesity	866.00 (11.49%)	296.00 (25.06%)	570.00 (8.97%)	
Heart disease, *n* (%)				< 0.001
No	6,649.00 (88.24%)	917.00 (77.65%)	5,732.00 (90.21%)	
Yes	886.00 (11.76%)	264.00 (22.35%)	622.00 (9.79%)	
Hypertension, *n* (%)				< 0.001
No	4502.00 (59.75%)	381.00 (32.26%)	4121.00 (64.86%)	
Yes	3033.00 (40.25%)	800.00 (67.74%)	2233.00 (35.14%)	
Dyslipidemia, *n* (%)				< 0.001
No	6809.00 (90.36%)	885.00 (74.94%)	5924.00 (93.23%)	
Yes	726.00 (9.64%)	296.00 (25.06%)	430.00 (6.77%)	
Stroke incident, *n* (%)	431.00 (5.72%)	111.00 (9.40%)	320.00 (5.04%)	< 0.001

Abbreviations: ABSI, a body shape index; BRI, body roundness index; CircS, circadian syndrome; DBP, diastolic blood pressure; FBG, fasting blood glucose; HbA1c, hemoglobin A1c; HDL‐C, high‐density lipoprotein cholesterol; LDL‐C, low‐density lipoprotein cholesterol; SBP, systolic blood pressure; TC, total cholesterol; TGs, triglycerides; VAI, visceral adiposity index; WHtR, waist‐to‐height ratio.

^a^Pearson′s chi‐squared test; Wilcoxon rank sum test.

In the study, the median age of participants was 59 years, with an age range of 52–65 years, and 46.9% were male. Compared with the no‐CircS group, which had a median age of 58.0 years (52.0, 65.0), the CircS group was older, with a median age of 60.0 years (54.0, 66.0) (*p* < 0.001). Moreover, the CircS group had a notably higher proportion of females, with 72.7% compared with 49.5% (*p* < 0.001). The marital status varied significantly, with 83.9% of those in the CircS group married and living with a spouse, in contrast to 88.7% in the no‐CircS group (*p* < 0.001). Educational attainment was lower in the CircS group, with 77.1% having completed junior high school or less, compared with 68.2% in the no‐CircS group (*p* < 0.001).

Regarding smoking and drinking status, significant differences were observed between the groups. Nonsmokers were more prevalent in the CircS group (74.09% vs. 57.95%), whereas current smoking was higher in the no‐CircS group (33.24% vs. 17.70%). Additionally, CircS participants were more likely to never drink (80.86% vs. 67.56%), whereas current drinkers were more common in the no‐CircS group (27.34% vs. 13.80%). The CircS group exhibited significantly higher prevalence of heart disease (22.35% vs. 9.79%), hypertension (67.74% vs. 35.14%), dyslipidemia (25.06% vs. 6.77%), and obesity (25.06% vs. 8.97%).

### 3.2. Association of BRI With Stroke Risk

Throughout the follow‐up duration, stroke events occurred in 431 participants, representing 5.7% of the total 7535. According to Table 2, each sequentially adjusted model consistently linked higher log (BRI) values with a greater risk of stroke. Model 3 showed that a one‐unit increment in log (BRI) was associated with a 2.50‐fold increase in stroke risk (HR = 2.50, 95% CI: 1.81–3.44, *p* < 0.001). Throughout Models 1–3, the positive correlation remained significant (Model 1: HR = 2.47; Model 2: HR = 2.78; Model 3: HR = 2.50; all *p* < 0.001).

**Table 2:** The HR (95% CI) of stroke according to BRI in the three models.

Characteristic	N	Event *N*	Model 1			Model 2			Model 3		
			HR	95% CI	*p*	HR	95% CI	*p*	HR	95% CI	*p*
Log (BRI)	7535	431	2.47	1.84, 3.32	< 0.001	2.78	2.02, 3.82	< 0.001	2.50	1.81, 3.44	< 0.001
Quartile	7535	431									
Q1	1884		Reference			Reference			Reference		
Q2	1884		1.32	0.96, 1.81	0.089	1.38	1.01, 1.91	0.046	1.36	0.99, 1.87	0.060
Q3	1883		2.02	1.51, 2.71	< 0.001	2.22	1.65, 3.00	< 0.001	2.11	1.56, 2.85	< 0.001
Q4	1884		2.17	1.63, 2.91	< 0.001	2.41	1.77, 3.28	< 0.001	2.23	1.63, 3.04	< 0.001
*p* *_for trend_*					< 0.001			< 0.001			< 0.001

*Note:* Model 1: unadjusted; Model 2: adjusted for age, gender, marital status, education level, drinking status, and smoking status; Model 3: Model 2 + adjusted for heart disease, hypertension, dyslipidemia, obesity status, serum creatinine, and blood urea nitrogen.

Abbreviations: BRI, body roundness index; CI, confidence interval; HR, hazard ratio.

Compared with individuals in the lowest BRI quartile (Q1), those in Q2 (HR = 1.36, 95% CI: 0.99–1.87, *p* = 0.060), Q3 (HR = 2.11, 95% CI: 1.56–2.85, *p* < 0.001), and Q4 (HR = 2.23, 95% CI: 1.63–3.04, *p* < 0.001) exhibited progressively higher stroke risks in Model 3. A significant trend was observed across the quartiles (*p* for trend < 0.001).

Figure 2a′s RCS analysis also confirmed that the connection between BRI and stroke risk is nearly linear (*p* for overall ≤ 0.001, *p* for nonlinear = 0.156), supporting the observed trend. Kaplan–Meier survival estimates for the total cohort showed a stepwise increase in cumulative stroke incidence across ascending BRI quartiles (log‐rank *p* < 0.0001; Figure 3a), with a clear and sustained divergence in survival curves that began early in the follow‐up period. Relative to Q1, Q4 demonstrated the highest incidence, succeeded by Q3 and Q2.

Figure 2Association between BRI and stroke risk across different CircS statuses, evaluated using a multivariable‐adjusted restricted cubic spline model. (a) Total participants; (b) participants with circadian syndrome (CircS); (c) participants without circadian syndrome (no‐CircS). Abbreviations: BRI, body roundness index; CircS, circadian syndrome; HR, hazard ratio; CI, confidence interval.

**Figure:**
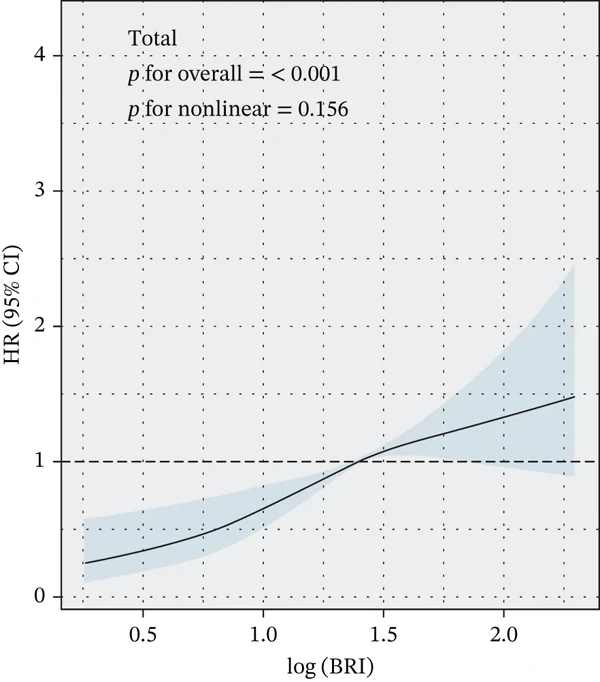
(a)

**Figure:**
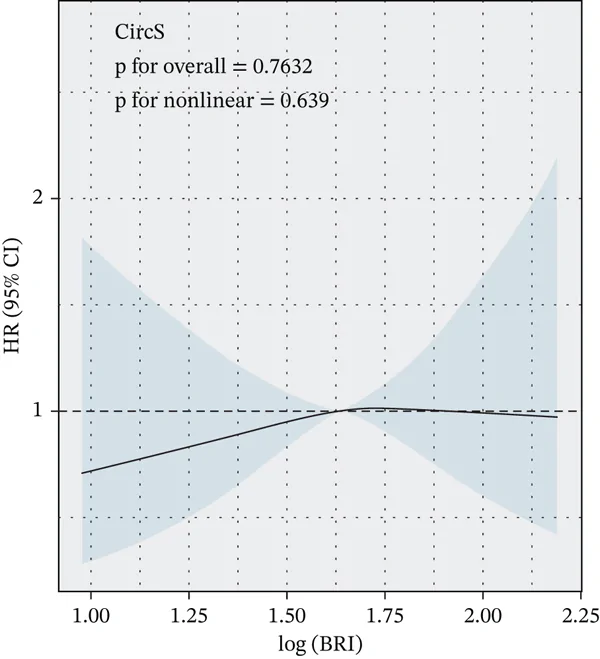
(b)

**Figure:**
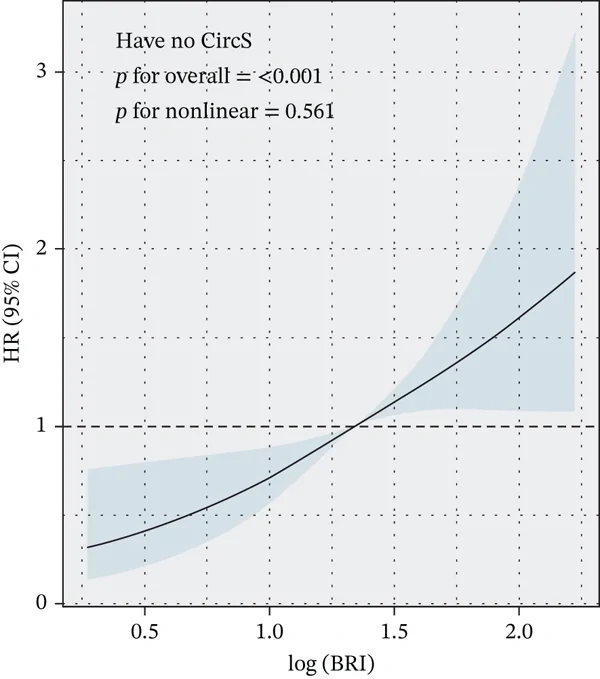
(c)

Figure 3Kaplan–Meier curves for stroke incidence across BRI quartiles, stratified by CircS status. (a) Total population; (b) participants with CircS; (c) participants without CircS. Abbreviations: BRI, body roundness index; CircS, circadian syndrome.

**Figure:**
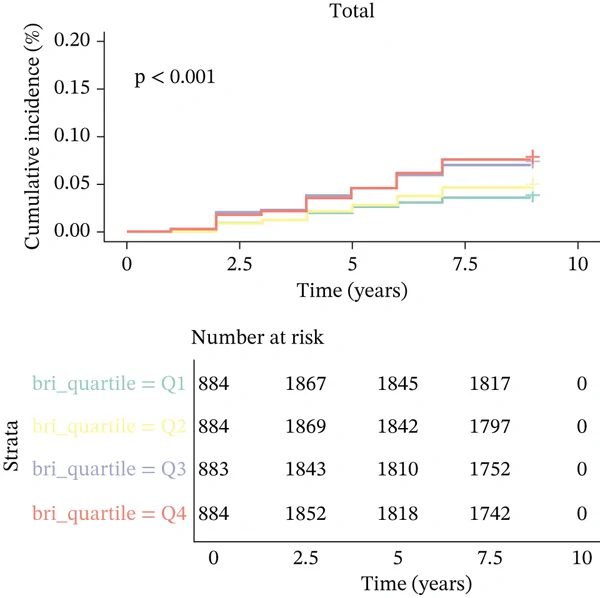
(a)

**Figure:**
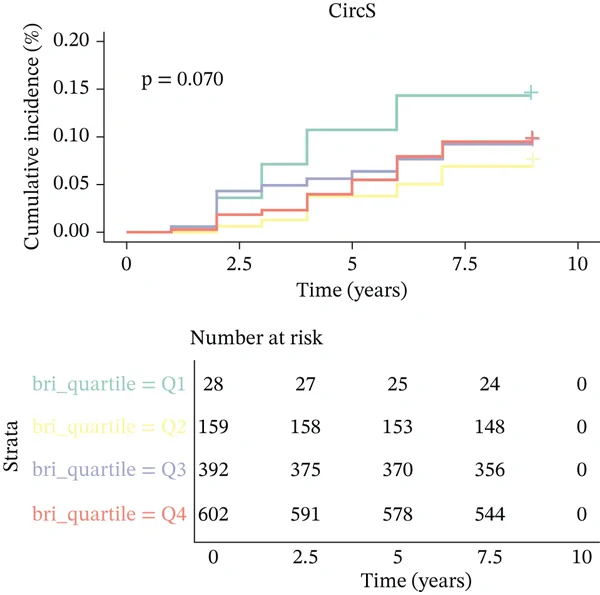
(b)

**Figure:**
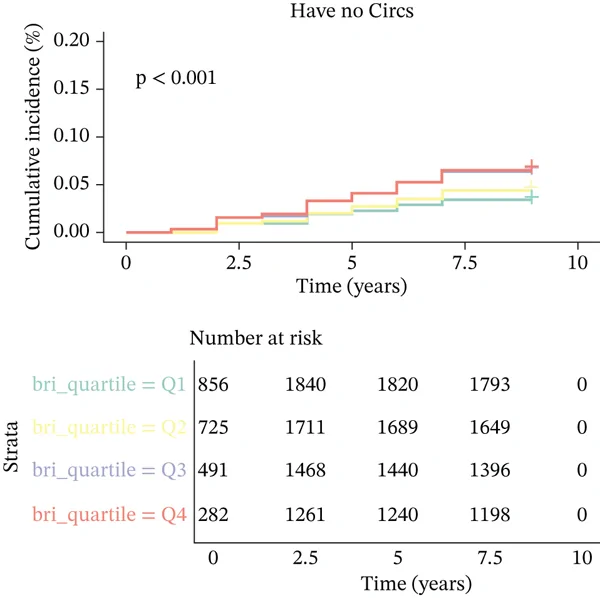
(c)

### 3.3. Association of BRI With Stroke Risk Under Different CircS Status

Table 3 displays the link between BRI and stroke risk across various CircS statuses. In the context of Model 3, adjusted for demographic and clinical factors, the relationship between log (BRI) and stroke was more pronounced in participants without CircS (HR = 2.36, 95% CI: 1.61–3.47, *p* < 0.001) compared with those with CircS (HR = 1.23, 95% CI: 0.55–2.76, *p* = 0.615). Consistent results were observed in the quartile‐based analysis. Among individuals without CircS, higher BRI quartiles were associated with progressively increased stroke risk (Q4 vs. Q1: HR = 2.08, 95% CI: 1.46–2.98, *p* < 0.001; *p* for trend < 0.001). In contrast, no clear dose‐response pattern was detected among participants with CircS (Q4 vs. Q1: HR = 0.61, 95% CI: 0.21–1.77, *p* = 0.363; *p* for trend = 0.879). Given the limited number of stroke events in the lowest BRI quartile among participants with CircS, quartile‐based estimates in this subgroup should be interpreted with caution.

**Table 3:** Association between BRI and the risk of stroke according to CircS status.

Characteristic	N	Event *N*	Model 1			Model 2			Model 3		
			HR	95% CI	*p*	HR	95% CI	*p*	HR	95% CI	*p*
CircS											
Log (BRI)	1181	111	1.21	0.57, 2.57	0.617	1.36	0.62, 2.99	0.440	1.23	0.55, 2.76	0.615
Quartile	1181	111									
Q1	28	4	Reference			Reference			Reference		
Q2	159	12	0.50	0.16, 1.54	0.226	0.50	0.16, 1.56	0.234	0.47	0.15, 1.48	0.195
Q3	392	37	0.64	0.23, 1.79	0.393	0.66	0.23, 1.87	0.433	0.61	0.21, 1.77	0.366
Q4	602	58	0.64	0.23, 1.78	0.396	0.68	0.24, 1.91	0.467	0.61	0.21, 1.77	0.363
*p* *_for trend_*					0.853			0.725			0.879
Have no CircS											
Log (BRI)	6354	320	2.26	1.60, 3.19	< 0.001	2.57	1.78, 3.71	< 0.001	2.36	1.61, 3.47	< 0.001
Quartile	6354	320									
Q1	1856	63	Reference			Reference			Reference		
Q2	1725	76	1.30	0.93, 1.82	0.121	1.37	0.98, 1.91	0.068	1.34	0.96, 1.88	0.086
Q3	1491	96	1.93	1.40, 2.65	< 0.001	2.12	1.54, 2.93	< 0.001	2.02	1.45, 2.81	< 0.001
Q4	1282	85	1.98	1.43, 2.75	< 0.001	2.23	1.58, 3.14	< 0.001	2.08	1.46, 2.98	< 0.001
*p* *_for trend_*					< 0.001			< 0.001			< 0.001

*Note:* Model 1: unadjusted; Model 2: adjusted for age, gender, marital status, education level, drinking status, and smoking status; Model 3: Model 2 + adjusted for heart disease, hypertension, dyslipidemia, obesity status, serum creatinine, and blood urea nitrogen.

Abbreviations: BRI, body roundness index; CI, confidence interval; CircS, circadian syndrome; HR, hazard ratio.

For those with CircS, the RCS model (Figure 2b) found no evidence of linear or nonlinear associations between BRI and stroke risk (overall *p* = 0.763; nonlinear *p* = 0.639). In contrast, individuals without CircS demonstrated a clear linear dose–response relationship, as indicated by the RCS analysis (Figure 2c; *p* for overall < 0.001; *p* for nonlinear = 0.561) Consistent with these findings, Kaplan–Meier survival curves stratified by BRI quartiles showed minimal variation in stroke incidence among CircS participants (Figure 3b; log‐rank *p* = 0.660), whereas a pronounced gradient in cumulative stroke risk was observed in individuals without CircS (Figure 3c; log‐rank *p* < 0.001).

### 3.4. Subgroup Analysis

Subgroup analyses that are aimed at identifying potential heterogeneity were stratified by sex, age groups, heart disease status, and lifestyle factors, using both log (BRI) values and BRI quartiles (Tables S3 and S4). The association was stronger in males than in females, with higher HRs per one‐unit increase in log (BRI) (HR: 2.95 vs. 1.61) and corresponding quartile comparisons (Q4 vs. Q1 HR: 2.13 vs. 1.81). Participants younger than 60 years exhibited more pronounced associations (log (BRI) HR: 3.33; Q4 vs. Q1 HR: 2.76). Lifestyle factors further modified the association. Alcohol consumption was associated with the greatest risk elevation (HR per one‐unit increase in log (BRI): 3.17; Q4 vs. Q1 HR: 2.30), whereas smoking was also associated with a significantly increased risk (Q4 vs. Q1 HR: 2.15). In addition, statistically significant associations were mainly observed among participants without pre‐existing heart disease (Q4 vs. Q1 HR: 2.13). All subgroup models were fully adjusted for demographic characteristics, behavioral risk factors, and clinical covariates. These findings overall imply that the connection between increased BRI and stroke risk could be more noticeable in younger males and people with negative lifestyle choices.

### 3.5. Sensitivity Analysis

Several sensitivity analyses were performed to evaluate the stability of the relationship between BRI and stroke risk. First, participants with missing data were excluded, and the relationship was reassessed. Even with this restriction, the fully adjusted model (Model 3) maintained a strong positive association between log (BRI) and stroke incidence (HR = 2.12, 95% CI: 1.51–2.96, *p* < 0.001), with a clear dose–response pattern across BRI quartiles (Q4 vs. Q1: HR = 1.96, 95% CI: 1.42–2.71, *p* < 0.001) (Table S5). By excluding stroke events in the first 2 years of follow‐up to mitigate reverse causality, a parallel analysis still found the association to be significant. In this analysis, the fully adjusted model showed similar findings [log (BRI): HR = 2.06, 95% CI: 1.46–2.90, *p* < 0.001; Q4 vs. Q1: HR = 1.90, 95% CI: 1.37–2.64, *p* < 0.001], as detailed in Table S6. These sensitivity analyses bolster the primary conclusions, proving that the association between increased BRI and stroke risk is consistent, no matter how missing data are addressed or early events are excluded.

### 3.6. The Predictive Role of Baseline BRI for Stroke Risk

**Figure 4:**
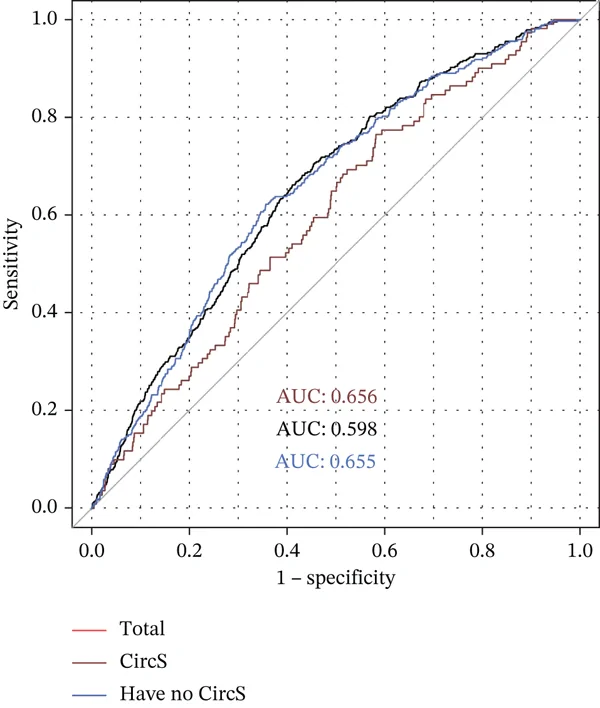
Discriminative performance of BRI for incident stroke prediction across CircS strata. Abbreviations: BRI, body roundness index; CircS, circadian syndrome; AUC, area under the curve; ROC, receiver operating characteristic.

**Figure 5:**
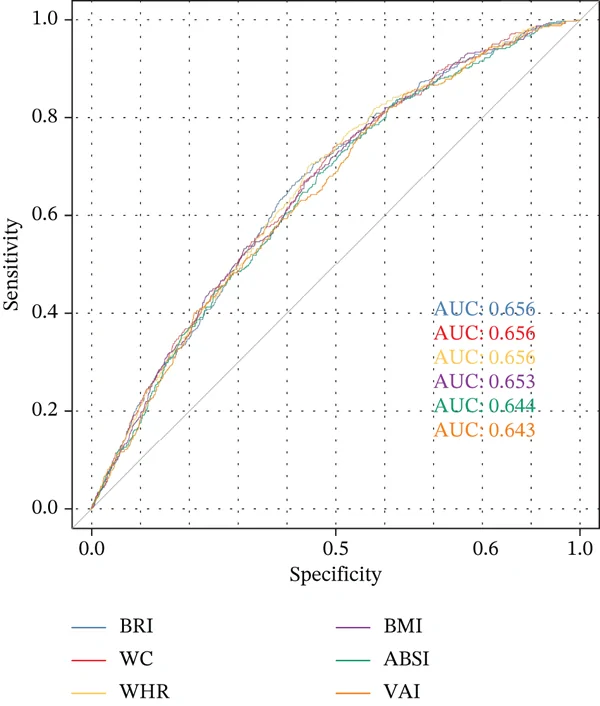
Comparative predictive performance of anthropometric indices for incident stroke. Abbreviations: BRI, body roundness index; WC, waist circumference; WHR, waist‐to‐hip ratio; BMI, body mass index; ABSI, A body shape index; VAI, visceral adiposity index; AUC, area under the curve; ROC, receiver operating characteristic.

The predictive capability of BRI for stroke incidence was assessed using ROC curve analyses in both the general population and subgroups categorized by CircS status. The AUC for BRI in the entire sample was 0.656 (95% CI: 0.631–0.681). The AUC was somewhat lower at 0.598 (95% CI: 0.545–0.651) for those with CircS, whereas individuals without CircS showed higher discriminative ability with an AUC of 0.655 (95% CI: 0.626–0.684) (Figure 4; Table 4). Moreover, ROC curve analyses were carried out to assess the discriminatory power of BRI against five other common anthropometric indices: BMI, WC, waist‐to‐hip ratio (WHR), a body shape index (ABSI), and the visceral adiposity index (VAI). As shown in Figure 5 and Table 4, BRI demonstrated good discriminatory ability with an AUC of 0.656 (95% CI: 0.631–0.681, *p* < 0.001). BRI′s AUC was comparable with WC and WHR and slightly higher than BMI (AUC = 0.653), ABSI (AUC = 0.644), and VAI (AUC = 0.643). The BRI cutoff point of 0.143 was optimal, providing a sensitivity of 70.8% (95% CI: 66.5%–75.1%) for recognizing individuals at risk.

**Table 4:** ROC curve for adjusting CircS with different indicators.

Characteristic	Cutoff	AUC	AUC.low	AUC.up	SEN	SEN.low	SEN.up	SPE	SPE.low	SPE.up	*p*
BRI	0.143	0.656	0.631	0.681	0.708	0.665	0.751	0.544	0.532	0.556	< 0.001
WC	−0.255	0.656	0.631	0.681	0.740	0.699	0.782	0.506	0.494	0.517	< 0.001
WHR	−0.281	0.656	0.632	0.681	0.703	0.660	0.746	0.555	0.544	0.567	< 0.001
BMI	−0.281	0.653	0.628	0.678	0.682	0.638	0.726	0.551	0.540	0.563	< 0.001
ABSI	−0.397	0.644	0.619	0.669	0.687	0.643	0.731	0.537	0.525	0.548	< 0.001
VAI	−0.462	0.643	0.618	0.668	0.770	0.731	0.810	0.449	0.437	0.460	< 0.001
Total	0.143	0.656	0.631	0.681	0.708	0.665	0.751	0.544	0.532	0.556	< 0.001
Circs	−0.861	0.598	0.545	0.651	0.766	0.687	0.845	0.419	0.389	0.448	< 0.001
Have no CircS	0.738	0.655	0.626	0.684	0.622	0.569	0.675	0.643	0.631	0.655	< 0.001

Abbreviations: ABSI, a body shape index; AUC, area under the curve; BMI, body mass index; BRI, body roundness index; CircS, circadian syndrome; SEN, sensitivity; SPE, specificity; VAI, visceral adiposity index; WC, waist circumference; WHR, waist‐to‐hip ratio.

### 3.7. Mediation Analysis

**Figure 6:**
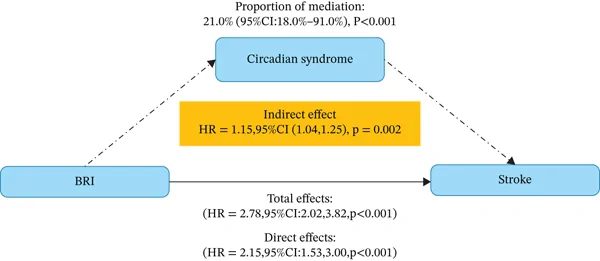
Mediation analysis of the association between BRI and stroke risk through CircS. Abbreviations: BRI, body roundness index; HR, hazard ratio; CI, confidence interval; CircS, circadian syndrome.

To grasp the mechanisms that associate BRI with stroke risk, a mediation analysis was carried out, involving both metabolic and behavioral elements (CircS conditions). Based on the mediation analysis results presented in Figure 6, the IE of CircS on stroke risk was significant, with a HR of 1.15 (95% CI: 1.04–1.25, *p* = 0.002). This mediation effect accounted for 21.0% of the total association between BRI and stroke risk (95% CI: 18%–91%, *p* < 0.001). The DE of CircS on stroke risk was also significant, with an HR of 2.15 (95% CI: 1.53–3.00, *p* < 0.001). Both the DE and IE remained significant across all models. We performed a sensitivity analysis by redefining CircS without the WC (central obesity) component (Figure S1). The DE of CircS on stroke risk was also significant, with an HR of 1.90 (95% CI: 1.23–3.85, *p* < 0.001). This mediation effect accounted for 20.30% of the total association between BRI and stroke risk (95% CI: 9.0%–45.6%, *p* < 0.001). The IE of CircS on stroke risk was significant, with an HR of 1.18 (95% CI: 1.04–1.23, *p* < 0.001).

## 4. Discussion

This large, nationally representative cohort of middle‐aged and older Chinese adults demonstrated a robust and consistent association between elevated BRI and an increased risk of stroke incidents. The findings were supported by the persistence of this association across various analytical strategies, including Cox regression, RCS modeling, Kaplan–Meier analysis, subgroup analyses, and sensitivity analyses. This study highlights that the association between BRI and stroke was notably different according to CircS status. For individuals without CircS, a higher BRI was strongly and linearly associated with an increased risk of stroke, with clear dose–response relationships observed in both continuous and quartile‐based analyses. In contrast, no significant association or dose–response gradient was observed among participants with CircS. These findings imply that the association between central adiposity and stroke risk may differ with CircS status, potentially reducing the predictive effectiveness of BRI in this subgroup. However, these results should be interpreted with caution. The limited occurrence of stroke events in the lowest BRI quartile among those with CircS could reduce the reliability and accuracy of quartile‐based estimates. In this context, the quartile analyses in the CircS subgroup are treated as exploratory and meant for hypothesis generation instead of confirmation. Importantly, the primary inference in this subgroup is supported by analyses treating BRI as a continuous variable, which avoids sparse reference categories and consistently demonstrates no significant association across all adjusted models, lending internal consistency to the overall findings. Mediation analysis further indicated that CircS may account for a meaningful proportion of the association between BRI and stroke risk, with approximately 21% of the TE operating through pathways related to circadian‐related metabolic dysregulation. Even though this analysis does not define temporal order or causality, it offers initial evidence that circadian health could act as an intermediary in the connection between central adiposity and stroke development. Altogether, these results highlight BRI as a straightforward and insightful anthropometric marker for determining stroke risk, especially in individuals without CircS. Moreover, they point out the necessity of considering circadian‐related metabolic conditions in the interpretation of cerebrovascular risks associated with obesity. In this comparative analysis, BRI was found to be as effective as WC and WHR, and slightly superior to BMI, ABSI, and VAI, indicating that BRI captures obesity‐related stroke risk at least as well as standard measures.

Our results further support existing evidence regarding the detrimental effects of abnormal fat distribution on cerebrovascular conditions. Previous investigations have consistently revealed that standard measures of adiposity, such as BMI, are linked to an increased risk of stroke [30], but the magnitude of this link varies across different populations [4]. A meta‐analysis by Strazzullo et al. revealed that each 5‐kg/m^2^ boost in BMI was connected to a 40% elevated risk of ischemic stroke [4]. Despite its use, BMI is unable to distinguish between visceral and subcutaneous fat, reducing its accuracy in evaluating metabolic risks linked to obesity [9]. In contrast, growing evidence suggests that novel anthropometric indices may better reflect central adiposity and its vascular consequences. The BRI, as proposed by Thomas et al., was created to provide a more accurate representation of body shape and visceral fat accumulation than BMI [6]. Subsequent research has affirmed its relevance in clinical practice. Liu et al. notably illustrated that BRI was superior to BMI and WC in predicting cardiovascular events within a Chinese population [9]. Our findings are in line with these results and further increase the predictive relevance of BRI for incident stroke in a large, nationally representative Chinese cohort [31].

Further stratified analyses by age, sex, and lifestyle factors indicated that the association between BRI and stroke risk was more pronounced among individuals younger than 60 years. This pattern may indicate a stronger association between adiposity and vascular health in these subgroups, consistent with previous studies [32, 33]. In addition, individuals with lifestyle risks like alcohol consumption faced a higher stroke risk when BRI was increased, suggesting a synergistic effect between behavioral and metabolic risk factors.

CircS′s modification of the effect is particularly notable. Initially, CircS was proposed as a composite measure reflecting circadian‐related metabolic dysregulation and has been independently linked to increased risks of stroke and cardiovascular mortality [13]. According to our knowledge, no study has previously analyzed the interaction between BRI and CircS status. To our knowledge, this is the first study to explore whether CircS modifies the association between BRI and stroke risk. One interpretation could be that visceral fat is a leading contributor to cerebrovascular risk in individuals who do not have CircS. In contrast, among those with CircS, the coexistence of multiple metabolic abnormalities may attenuate the apparent contribution of adiposity alone [27]. Circadian disruption‐related metabolic disturbances may obscure the incremental impact of body shape on stroke risk in these contexts. In summary, the data suggest that CircS may change the association between body fat and cerebrovascular outcomes, possibly through metabolic pathways that independently raise the risk of stroke.

Mechanistically, our mediation analysis suggests that CircS plays a significant mediating role in the relationship between BRI and stroke risk. CircS was proposed as a comprehensive construct of cardiometabolic risk, incorporating the key metabolic syndrome elements with circadian‐related behavioral disturbances. Operationally, CircS is defined by the presence of at least four of seven co‐occurring components, namely central obesity, elevated TGs, reduced high‐density lipoprotein cholesterol, elevated blood pressure, impaired fasting glucose, short sleep duration, and depressive symptoms. This clustered phenotype captures the cumulative burden of metabolic dysregulation together with circadian disruption, which may plausibly promote cerebrovascular vulnerability through converging pathways involving neuroendocrine imbalance, chronic low‐grade inflammation, and accelerated atherogenesis [34–37]. In this context, CircS can be viewed as a system‐level indicator that partially transmits the adverse vascular consequences of central adiposity, rather than reflecting the effect of any single abnormality [25, 37]. Notwithstanding this mechanistic plausibility, an important methodological issue warrants consideration. Since CircS, as originally specified, includes a central obesity component based on WC, and BRI is mathematically derived from WC and height, a degree of structural overlap exists between the exposure and mediator. This definitional proximity introduces the possibility that part of the observed IE may arise from shared anthropometric information rather than from a fully distinct biological pathway. To evaluate this concern, we conducted sensitivity analyses in which CircS was redefined after excluding the WC component, and the mediation models were re‐estimated under this alternative specification. The IE retained its statistical significance and directional consistency, even though its magnitude was modestly lessened. These findings suggest that the mediation pathway is not exclusively due to shared measurement structure and provide additional backing for the consistency of the observed association. Nonetheless, careful consideration is necessary when interpreting causality. Adiposity‐related indices inherently capture overlapping physiological dimensions, and complete statistical disentanglement of these constructs may be difficult in observational analyses. Accordingly, our findings should be interpreted as supporting a potential mediating role of circadian‐related cardiometabolic dysregulation within an adiposity‐linked risk framework, rather than as conclusive evidence of an independent causal mechanism.

During ROC analyses, BRI displayed moderate discriminative power for predicting incident stroke, with an AUC of about 0.65. The study′s primary purpose was to examine the association between BRI and stroke risk, rather than to design a clinical prediction model. With the complex nature of stroke, it is unsurprising that a single anthropometric measure provides only modest discrimination. Importantly, ROC analyses were conducted mainly to compare the relative performance of BRI with other commonly used anthropometric indices. BRI performed on par with WC and WHR, and slightly surpassed BMI, ABSI, and VAI, suggesting that it captures obesity‐related stroke risk as effectively as traditional measures. The focus on the BRI is due to its improved capacity to reflect central adiposity in comparison to traditional anthropometric metrics. Unlike BMI, which reflects overall body mass without accounting for fat distribution, BRI integrates WC and height to approximate body shape and abdominal fat accumulation, which are more closely linked to cardiometabolic dysfunction and vascular risk. The presence of central adiposity has been consistently associated with the development of insulin resistance, systemic inflammation, and atherosclerosis, all of which are critical pathways to stroke [38–41]. In this context, limited discriminative performance is not unexpected, as stroke risk arises from the interplay of multiple metabolic, vascular, and behavioral factors beyond adiposity alone. Accordingly, the observed AUC values should be interpreted in terms of relative comparative performance rather than absolute predictive accuracy. Overall, the moderate AUC values point to the limitations of using a single variable for prediction, without detracting from the strong and consistent associations between BRI and stroke risk.

Yet, several limitations should be recognized. First, participants′ self‐reported physician diagnoses were used to identify initial stroke incidents, rather than clinical adjudication or medical record validation, which may introduce errors in outcome classification. Nonetheless, this misclassification is probably nondifferential concerning BRI, which would likely skew effect estimates towards the null rather than exaggerate observed associations. Second, despite BRI being a stand‐in marker for central adiposity, visceral fat was not directly quantified using imaging tools, which might have provided clearer mechanistic insights into the observed connections. Third, given that the study population was exclusively Chinese middle‐aged and older adults, it is important to be cautious when extending these results to other ethnic or geographic groups. To further clarify the complex relationship between adiposity, CircS, and stroke risk, future studies should include objective assessments of circadian rhythms and imaging‐based measures of fat distribution.

## 5. Conclusion

Within this nationwide cohort of middle‐aged and older Chinese adults, a higher BRI was consistently associated with a heightened risk of stroke. The association was consistently strong across various analytical strategies and was mainly evident in individuals without CircS, while it was lessened in those with CircS. Mediation analysis indicated that CircS may explain part of the BRI‐stroke association, suggesting a potential role of circadian‐related metabolic dysregulation. Overall, these findings endorse BRI as a straightforward anthropometric tool for stroke risk assessment and underline the importance of circadian health in managing cerebrovascular risk associated with obesity.

## Author Contributions

The study design, data collection, analysis, and interpretation were equally contributed to by G.M., H.L., H.W., M.Z., Y.Z., H.F., and K.S. (all authors). K.S. and H.F. contributed equally to this work and should be considered as co‐corresponding authors.

## Funding

This study was supported by Shanxi Health Committee Foundation Project (2024057); Fundamental Research Program of Shanxi Province (202403021222432); and Key Research and Development Program of the Shanxi Provincial Department of Science and Technology (202102020101009).

## Disclosure

All authors were involved in drafting, revising, and approving the final manuscript.

## Ethics Statement

The ethical guidelines of the Declaration of Helsinki were followed in all procedures. As the study used de‐identified data from publicly available sources, it did not require further ethical review and does not meet the criteria for a prospective study needing ethical approval.

## Consent

The authors have nothing to report.

## Conflicts of Interest

The authors declare no conflicts of interest.

## Supporting information


**Supporting Information** Additional supporting information can be found online in the Supporting Information section. Figure S1: Mediation analysis of the association between BRI and stroke risk through CircS, excluding waist circumference. Table S1: Distribution of missing data. Table S2: Variance inflation factor analysis of clinical covariates. Table S3: Subgroup analysis of the association between log (BRI) and stroke. Table S4: Subgroup analysis of the association between BRI (quartile increase) and stroke. Table S5: The HR (95% CI) of stroke according to BRI in the three models with missing data. Table S6: The HR (95% CI) of stroke according to BRI in the three models, excluding strokes occurring within the first 2 years.

## Data Availability

The data that support the findings of this study are available from the corresponding authors upon reasonable request.
