# Supplementary material for: Circadian Syndrome Mediates the Association Between Body Roundness Index and Incident Stroke Risk in Middle‐Aged and Older Chinese Adults: A Nationwide Prospective Cohort Study
Source: Behav Neurol. 2026 Mar 27;2026:1282354. doi: 10.1155/bn/1282354 (PMC13580791; doi:10.1155/bn/1282354)
Supplement: Supplementary file 1 [file BN-2026-1282354-s001.docx]

**Circadian syndrome mediates the association between body roundness index and incident stroke risk in middle-aged and older Chinese adults: a nationwide prospective cohort study**

**Supplementary Figure 1** Mediation analysis of the association between BRI and stroke risk through CircS, excluding waist Circumference

**Supplementary Table1** Distribution of missing data

**Supplementary Table 2** Variance inflation factor analysis of clinical covariates

**Supplementary Table 3** Subgroup analysis of the association between log (BRI) and stroke

**Supplementary Table 4** Subgroup analysis of the association between BRI (quatile increase) and stroke

**Supplementary Table 5** The HR (95% CI) of stroke according to BRI in the three models with missing data

**Supplementary Table 6** The HR (95%CI) of stroke according to BRI in the three models, excluding strokes occurring within the first two years

**Supplementary Figure 1** Mediation analysis of the association between BRI and stroke risk through CircS, excluding waist Circumference

**Abbreviations**: BRI, body roundness index; HR, hazard ratio; CI, confidence interval; CircS, circadian syndrome

**Supplementary Table1** Distribution of missing data

| **Characteristic** | **No. of missing values** | **Percent(%)** |
| --- | --- | --- |
| **BRI** | 0 | 0.00 |
| **Age** | 0 | 0.00 |
| **Gender** | 0 | 0.00 |
| **SBP** | 77 | 1.02 |
| **DBP** | 93 | 1.23 |
| **Marital status** | 0 | 0.00 |
| **Education level** | 0 | 0.00 |
| **Smoking status** | 1 | 0.01 |
| **Drinking status** | 1 | 0.01 |
| **Heart rate** | 52 | 0.69 |
| **Heart disease** | 22 | 0.29 |
| **Hypertension** | 0 | 0.00 |
| **Dyslipidemia** | 69 | 0.92 |
| **WC** | 0 | 0.00 |
| **WHR** | 0 | 0.00 |
| **TC** | 3 | 0.04 |
| **TG** | 0 | 0.00 |
| **HDL** | 0 | 0.00 |
| **LDL** | 15 | 0.20 |
| **HbA1c** | 56 | 0.74 |
| **FBG** | 11 | 0.15 |
| **CREA** | 16 | 0.21 |
| **BUN** | 2 | 0.03 |
| **Obesity** | 17 | 0.23 |
| **ABSI** | 0 | 0.00 |
| **VAI** | 17 | 0.00 |

**Abbreviation:** ABSI, a body shape index; BRI, body roundness index; CircS, circadian syndrome; DBP, diastolic blood pressure; FBG, fasting blood glucose; HbA1c, hemoglobin A1c; HDL-C, high-density lipoprotein cholesterol; LDL-C, low-density lipoprotein cholesterol; SBP, systolic blood pressure; TC, total cholesterol; TG, triglycerides; VAI, visceral adiposity index; WHtR, waist-to-height ratio

**Supplementary Table 2** Variance inflation factor analysis of clinical covariates

| **Characteristics** | **VIF** |
| --- | --- |
| **Age, (years)** | 2.4 |
| **Gender** | 1.1 |
| **Marital status** | 1.4 |
| **education level** | 1.6 |
| **Drinking status** | 2.1 |
| **Smoking status** | 2.1 |
| **Heart disease** | 1.3 |
| **Hypertension** | 1.2 |
| **Dyslipidemia** | 1.1 |
| **Obesity status** | 2.6 |
| **Serum creatinine** | 1.2 |
| **Blood urea nitrogen** | 1.1 |

**Supplementary Table 3** Subgroup analysis of the association between log (BRI) and stroke

| **Characteristics** | **N** | **Event N** | **HR** | **95%CI** | **p-value** | **p-for_interaction_** |
| --- | --- | --- | --- | --- | --- | --- |
| **Gender** |  |  |  |  |  | 0.045 |
| Male | 3,534 | 210 | 2.95 | 1.79, 4.86 | <0.001 |  |
| Female | 4,001 | 221 | 1.61 | 1.03, 2.51 | 0.037 |  |
| **Age** |  |  |  |  |  | 0.032 |
| ≥60 | 3,433 | 234 | 1.59 | 1.03, 2.46 | 0.037 |  |
| <60 | 4,102 | 197 | 3.33 | 1.97, 5.63 | <0.001 |  |
| **Heart disease** |  |  |  |  |  | 0.074 |
| No | 6,649 | 345 | 2.45 | 1.67, 3.59 | <0.001 |  |
| Yes | 886 | 86 | 1.42 | 0.71, 2.86 | 0.323 |  |
| **Drinking status** |  |  |  |  |  | 0.027 |
| Never drunk | 5,248 | 288 | 1.79 | 1.20, 2.68 | 0.005 |  |
| Currently drinking or ever drunk | 2,287 | 143 | 3.17 | 1.74, 5.77 | <0.001 |  |
| **Smoking status** |  |  |  |  |  | 0.091 |
| Never smoked | 4,557 | 241 | 1.75 | 1.12, 2.74 | 0.014 |  |
| Currently smoking or ever smoked | 2,978 | 190 | 2.86 | 1.71, 4.76 | <0.001 |  |

**Abbreviation:** HR, hazard ratio; CI, confidence interval; BRI, body roundness index

**Supplementary Table 4** Subgroup analysis of the association between BRI (quatile increase) and stroke

| **Characteristics** | **N** | **Event N** | **HR** | **95%CI** | **p-value** |
| --- | --- | --- | --- | --- | --- |
| **Male** | 3,534 | 210 |  |  |  |
| Q1 | 1,256 |  | Reference | |  |
| Q2 | 1,019 |  | 1.16 | 0.77, 1.74 | 0.479 |
| Q3 | 811 |  | 2.14 | 1.46, 3.14 | <0.001 |
| Q4 | 448 |  | 2.13 | 1.37, 3.32 | <0.001 |
| *P _for trend_* |  |  |  |  | <0.001 |
| **Female** | 4,001 | 221 |  |  |  |
| Q1 | 628 |  | Reference | |  |
| Q2 | 865 |  | 1.52 | 0.88, 2.63 | 0.132 |
| Q3 | 1,072 |  | 1.68 | 1.00, 2.84 | 0.051 |
| Q4 | 1,436 |  | 1.81 | 1.08, 3.00 | 0.023 |
| *P _for trend_* |  |  |  |  | 0.032 |
| **≥60** | 3,433 | 234 |  |  |  |
| Q1 | 833 |  | Reference | |  |
| Q2 | 800 |  | 1.23 | 0.81, 1.87 | 0.340 |
| Q3 | 818 |  | 1.62 | 1.08, 2.42 | 0.020 |
| Q4 | 982 |  | 1.52 | 1.00, 2.32 | 0.049 |
| *P _for trend_* |  |  |  |  | 0.031 |
| **<60** | 4,102 | 197 |  |  |  |
| Q1 | 1,051 |  | Reference | |  |
| Q2 | 1,084 |  | 1.46 | 0.89, 2.41 | 0.136 |
| Q3 | 1,065 |  | 2.52 | 1.57, 4.06 | <0.001 |
| Q4 | 902 |  | 2.76 | 1.67, 4.55 | <0.001 |
| *P _for trend_* |  |  |  |  | <0.001 |
| **Heart disease (No)** | 6,649 | 345 |  |  |  |
| Q1 | 1,716 |  | Reference | |  |
| Q2 | 1,692 |  | 1.32 | 0.93, 1.89 | 0.121 |
| Q3 | 1,652 |  | 2.03 | 1.45, 2.84 | <0.001 |
| Q4 | 1,589 |  | 2.13 | 1.49, 3.05 | <0.001 |
| *P _for trend_* |  |  |  |  | <0.001 |
| **Heart disease (Yes)** | 886 | 86 |  |  |  |
| Q1 | 168 |  | Reference | |  |
| Q2 | 192 |  | 1.18 | 0.56, 2.49 | 0.671 |
| Q3 | 231 |  | 1.55 | 0.76, 3.19 | 0.230 |
| Q4 | 295 |  | 1.41 | 0.68, 2.93 | 0.351 |
| *P _for trend_* |  |  |  |  | 0.307 |
| **Never drunk** | 5,248 | 288 |  |  |  |
| Q1 | 1,149 |  | Reference | |  |
| Q2 | 1,251 |  | 1.55 | 1.02, 2.35 | 0.040 |
| Q3 | 1,336 |  | 1.99 | 1.33, 2.99 | <0.001 |
| Q4 | 1,512 |  | 1.90 | 1.25, 2.88 | 0.003 |
| *P _for trend_* |  |  |  |  | 0.003 |
| **Currently drinking or ever drunk** | 2,287 | 143 |  |  |  |
| Q1 | 735 |  | Reference | |  |
| Q2 | 633 |  | 1.02 | 0.61, 1.71 | 0.940 |
| Q3 | 547 |  | 1.93 | 1.20, 3.10 | 0.006 |
| Q4 | 372 |  | 2.30 | 1.37, 3.87 | 0.002 |
| *P _for trend_* |  |  |  |  | <0.001 |
| **Never smoked** | 4,557 | 241 |  |  |  |
| Q1 | 818 |  | Reference | |  |
| Q2 | 1,048 |  | 1.43 | 0.87, 2.35 | 0.155 |
| Q3 | 1,226 |  | 1.78 | 1.11, 2.85 | 0.017 |
| Q4 | 1,465 |  | 1.87 | 1.17, 3.00 | 0.009 |
| *P _for trend_* |  |  |  |  | 0.008 |
| **Currently smoking or ever smoked** | 2,978 | 190 |  |  |  |
| Q1 | 1,066 |  | Reference | |  |
| Q2 | 836 |  | 1.21 | 0.79, 1.85 | 0.392 |
| Q3 | 657 |  | 2.20 | 1.47, 3.29 | <0.001 |
| Q4 | 419 |  | 2.15 | 1.35, 3.42 | 0.001 |
| *P _for trend_* |  |  |  |  | <0.001 |

**Abbreviation:** HR, hazard ratio; CI, confidence interval; BRI, body roundness index

**Supplementary Table 5** The HR (95% CI) of stroke according to BRI in the three models with missing data

| **Characteristic** | **N** | **Event N** | **Model 1** | | | **Model 2** | | | **Model 3** | | |
| --- | --- | --- | --- | --- | --- | --- | --- | --- | --- | --- | --- |
|  |  |  | **HR** | **95%CI** | **p-value** | **HR** | **95%CI** | **p-value** | **HR** | **95%CI** | **p-value** |
| **Log (BRI)** | 7,535 | 431 | 2.47 | 1.84, 3.32 | <0.001 | 2.75 | 2.00, 3.78 | <0.001 | 2.12 | 1.51, 2.96 | <0.001 |
| **Quartile** | 7,535 | 431 |  |  |  |  |  |  |  |  |  |
| Q1 | 1,884 |  | Reference | |  | Reference | |  | Reference | |  |
| Q2 | 1,884 |  | 1.32 | 0.96, 1.81 | 0.089 | 1.37 | 0.99, 1.88 | 0.056 | 1.32 | 0.95, 1.82 | 0.094 |
| Q3 | 1,883 |  | 2.02 | 1.51, 2.71 | <0.001 | 2.19 | 1.63, 2.95 | <0.001 | 1.99 | 1.46, 2.70 | <0.001 |
| Q4 | 1,884 |  | 2.17 | 1.63, 2.91 | <0.001 | 2.38 | 1.76, 3.23 | <0.001 | 1.96 | 1.42, 2.71 | <0.001 |
| *P _for trend_* |  |  |  |  | <0.001 |  |  | <0.001 |  |  | <0.001 |

**Model 1**: unadjusted; **Model 2**: adjusted for age, gender, marital status, education level**,** drinking status, smoking status; **Model 3**: Model 2+adjusted for heart disease, hypertension, dyslipidemia, obesity status, serum creatinine, blood urea nitrogen

**Abbreviation:** HR, hazard ratio; CI, confidence interval; BRI, body roundness index

**Supplementary Table 6** The HR (95%CI) of stroke according to BRI in the three models, excluding strokes occurring within the first two years

| **Characteristic** | **N** | **Event N** | **Model 1** | | | **Model 2** | | | **Model 3** | | |
| --- | --- | --- | --- | --- | --- | --- | --- | --- | --- | --- | --- |
|  |  |  | **HR** | **95%CI** | **p-value** | **HR** | **95%CI** | **p-value** | **HR** | **95%CI** | **p-value** |
| **Log (BRI)** | 7,516 | 412 | 2.44 | 1.80, 3.30 | <0.001 | 2.67 | 1.93, 3.69 | <0.001 | 2.06 | 1.46, 2.90 | <0.001 |
| **Quartile** | 7,516 | 412 |  |  |  |  |  |  |  |  |  |
| Q1 | 1,882 |  | Reference | |  | Reference | |  | Reference | |  |
| Q2 | 1,881 |  | 1.31 | 0.95, 1.81 | 0.099 | 1.37 | 0.99, 1.90 | 0.056 | 1.31 | 0.94, 1.81 | 0.107 |
| Q3 | 1,875 |  | 1.96 | 1.45, 2.65 | <0.001 | 2.13 | 1.57, 2.90 | <0.001 | 1.87 | 1.37, 2.56 | <0.001 |
| Q4 | 1,878 |  | 2.15 | 1.60, 2.89 | <0.001 | 2.33 | 1.71, 3.19 | <0.001 | 1.90 | 1.37, 2.64 | <0.001 |
| *P _for trend_* |  |  |  |  | <0.001 |  |  | <0.001 |  |  | <0.001 |

**Model 1**: unadjusted; **Model 2**: adjusted for age, gender, marital status, education level**,** drinking status, smoking status; **Model 3**: Model 2+adjusted for heart disease, hypertension, dyslipidemia, obesity status, serum creatinine, blood urea nitrogen

**Abbreviation:** HR, hazard ratio; CI, confidence interval; BRI, body roundness index
